# Stable and reproducible expression of bacterial *ipt* gene under the control of SAM-specific promoter (pKNOX1) with interference of developmental patterns in transgenic *Peperomia pellucida* plants

**DOI:** 10.3389/fpls.2022.984716

**Published:** 2022-09-27

**Authors:** Phapawee Worakan, Ranjit Singh Gujjar, Kanyaratt Supaibulwatana

**Affiliations:** ^1^ Faculty of Science, Mahidol University, Rama VI Rd., Bangkok, Thailand; ^2^ Division of Crop Improvement, Indian Institute of Sugarcane Research, Lucknow, India

**Keywords:** *Peperomia pellucida*, pKNOX1, SAM-specific promoter, *ipt* gene, cytokinin

## Abstract

Reproducible and stable transgene expression is an important goal in plant basic research and applications. Hence, we report the first stable expression of bacterial transgenes in medicinal plant, *Peperomia pellucida* (L.) Kunth. Two key elements relevant to the dynamic expression of the bacterial cytokinin biosynthesis gene, *ipt* (isopentenyltransferase) were examined. First, by designing a specific expression cassette driven by a tissue-specific promoter for the required levels of gene expression in the particular function of development, and second by using *P. pellucida* as a model plant due to its short developmental cycle that supported expedient tracking of transgene expression in the progeny. Transgenic frequencies of *ipt* gene obtained from different expression cassettes of pKNOX1 for tissue-specific promoter in shoot apical meristem were compared with the cauliflower mosaic virus (CaMV35S) promoter (p35S), a constitutive promoter investigated for T_3_ generation. It was clearly shown that transgenic plants with pKNOX1 showed percentage of survivals in T_3_ at about 2.2 folds more than those of p35S-transgenic. Transgenic *P. pellucida* under controllable expression of pKNOX1 showed increased leaf and seed size with a high percentage of fertile seed, whereas transgenic plants with p35S showed phenotypic features of bushy and small leaves, sterile pollen and lower reproductive fitness. Quantitative examination of *ipt*-positive gene expression in T_3_ generation of transformants with pKNOX1 were 100% (line k-14) and 50% (line k-20), while 33.3% was observed in transgenic line c-11 with p35S. Interestingly, the endogenous cytokinin biosynthesis gene (*ipt3*) was significantly upregulated (2-3 folds higher) in pKNOX1-transformants. The overall relative mRNA expression of bacterial *ipt* gene and overproducing of cytokinin contents (t-ZR and 2-iP) detected in p35S-transformants caused abnormality and low percentages of transgene reproducible Interestingly, pKNOX1-transgenic plants tended to maintain chlorophyll contents 4-5 folds and extending the developmental cycle to 12.4 weeks (wk), which was 2 folds more than wildtype (5.8 wk) and p35S-transformants (7.4 wk). The promotor effect on stable and reproducible transgene-expressions demonstrated prominent features of *P. pellucida* and also empowered further omics studies.

## Graphical Abstract

The infographic abstract of the article “Stable and reproducible expression of bacterial ipt gene under the controlled of SAM-specific promoter (pKNOX1) with interference of developmental patterns in transgenic Peperomia pellucida plants”.
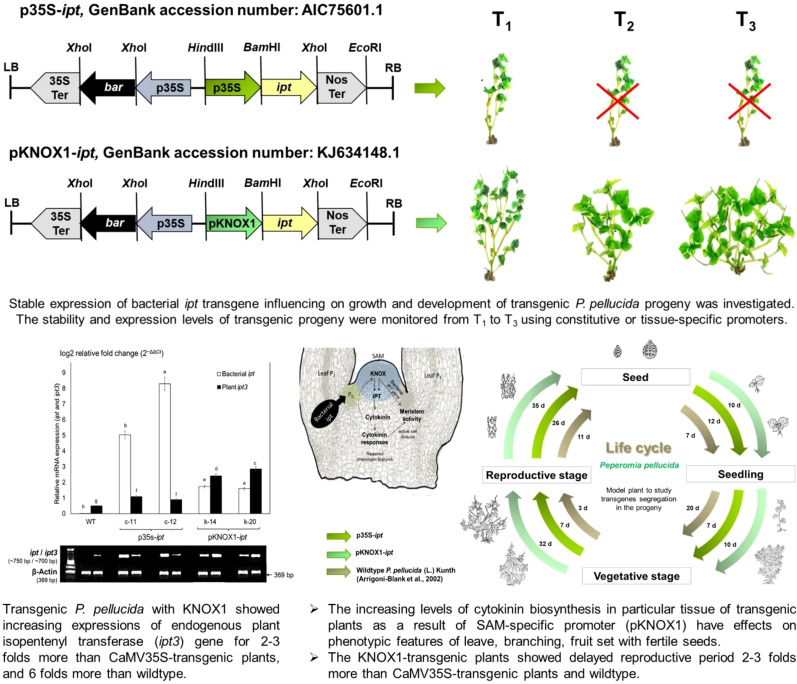

## Introduction

The bottleneck of plant gene transfer and segregation of transgenes in the progeny has been reported for a number of significant factors such as genotypic effect and the ability to regenerate next transgenic progeny (Atkins and Voytas, 2020). This includes the elements of T-DNA cassette design for effective delivering of target gene(s) to plant cells and efficient plasmid for *Agrobacterium*-mediated transformation (Lacroix and Citovsky, 2019), nevertheless it must not generate genomic shocks in host genome to avoid further gene suppression in plant progeny. Successive plant regeneration and monitoring of phenotypic responses at different growth and developmental stages of transgenic progenies are involved by actions of different phytohormone. Assessing hypothetical roles of cytokinin in plant growth and development using heterologous gene transfer of bacterial *ipt* gene driven by different promoters to interfere the levels of endogenous cytokinin biosynthesis in host plant and further examine the segregation of transgene through the progenies has been investigated in this present study. Cytokinin is an important phytohormone controlling several morphological developments such as stimulation of lateral growth, shoot branching and axillary bud outgrowth and physiological processes in terms of regulation of source-sink relationship (Wu et al., 2021), delay senescence and phytohormone crosstalk in cellular metabolic pathways (Nguyen et al., 2021). The cytokinin biosynthesis gene, *ipt* encodes a key enzyme (Akiyoshi et al., 1984), adenosine phosphate Isopentenyltransferase (IPT) that catalyzes conversion of the intermediate in the terpenoid biosynthesis pathway, isopentenyl-adenosine-5-monophosphate (DMAPP) and its isomer, isopentenyl diphosphate (IPP) to zeatin that is an active form of endogenous cytokinin in plants (Kakimoto, 2003).

Previous report showed the evidence of morphological and growth abnormalities in the transgenic plant that overexpressed with bacterial *ipt* genes to increase cytokinin contents in tobacco transgenic plants. The hyperbranching abnormalities, wrinkled curling leaves and dwarf shoots as well as problem of the sterility in tobacco reproductive systems were detected. The heterologous transgene expression of *ipt* in tobacco transformant was controlled by CAMV35S promoter (p35S), which revealed for a strong constitutive promoter resulted in overproduction of endogenous cytokinin and exhibited the retarded phenotype. Therefore, the fusion of *ipt* gene with intron were designed in the constructive T-DNA region in order to minimize the strong effect of p35S (Nawiri et al., 2018). Therefore, the constructional design of gene cassette under the control of appropriate promoter for *ipt* can functionally control cytokinin activities in transgenic tissues that may not cause abnormality in growth and development and lead to the stable and reproducible of transgene in the progenies of transgenic features. Specific responsive promoter constructs such as tissue-specific promoters and developmental process-related promoters expressed the *ipt* gene in a more controlled manner and limited the damage caused by engineering stress (Van et al., 1993). Hence, in this present work the promoter sequence of the *KNOX1* gene was used as a specific promoter to express *ipt* gene in the shoot apical meristematic (SAM) region to stimulate gene expression and cytokinin activities directly to promote plant growth and development of aerial parts in comparison with those control by p35S.

Transcription factor class I KNOX (KNOTTED-1-like HOMEOBOX) family genes regulate the formation and maintenance of shoot apical meristem development (Hake et al., 2004) and express important essential regulators for modulating the concentrations and activities of cytokinin. The expression pattern of KNOX1 is specifically limited in the organizing center (OC) zone of SAM, which governs active cell proliferation under high concentration of cytokinin (Zhang et al., 2021). The KNOX-cytokinin regulatory module in SAM which is a specific target area induces the appropriate expression of the *ipt* gene and led to spontaneous shoot formation (Meng et al., 2020).

One of key important factors is a plant model system that capable to support the fast and efficient tracking of stable segregation and reproducible transgene in transgenic progeny. *Peperomia pellucida* (L.) Kunth, a herbaceous plant widespread in the tropics and Asian countries (Kiew, 1999) is suggested to be used as a plant model in this study. *P. pellucida* plant has a complete life cycle within 37 days (Arrigoni-Blank et al., 2002), which is about 5-6 weeks whereas *Arabidopsis thaliana* requires about 8-12 weeks (Kumar, 2014). Moreover, *P. pellucida* is easy to grow and capable to produce seeds for both *in vitro* and *ex vitro* propagation. The *P. pellucida* plant also provides a good model to follow up the phenotypic expressions at different levels of morphological growth and development in responses to the external environments. This plant contains several phytochemical compounds, and the most common main substances are terpenoids that share the intermediate constituents in the mevalonate (MVA) and methylerythritol phosphate (MEP) biosynthetic pathways with cytokinin biosynthesis. We demonstrate here the attractive model of *P. pellucida* that efficiently provides further investigation of transgenic features in terms of transcriptomic, proteomic and metabolomic approaches. In addition, *P. pellucida* is a medicinal herb contains useful bioactive compounds that is very attractive for further study to improve the bioactive ingredients for pharmaceutical applications.

## Materials and methods

### Plasmid construction in *E. coli* and *Agrobacterium* containing bacterial *ipt* gene with constitutive (CaMV35S) and tissue-specific (KNOX1) promoters

The recipient plasmid was designed using pCAMBIA3300 (CAMBIA, Australia) as backbone. T-DNA fragment was designed using CaMV35S-*ipt* (2948 bp) that harboring a bacterial isopentenyl transferase (*ipt*) gene (GenBank accession number: P94207) under the control of the cauliflower mosaic virus CaMV35S promoter (Christou and Ford, 1995). The gene cassettes in T-DNA fragments were then modified by adding NOS terminator (GenBank accession number: AJ007624.1) and *bar* gene (GenBank accession number: X05822.1) encoding for phosphinothricin acetyltransferase as selectable marker gene (GenBank accession number: AIC75601.1). The recombinant plasmid was transformed into *E*. *coli* competent cell strain TOP10 (Invitrogen Cat no. C4040-10) by heat-shock transformation and cultured for blue-white screening at 37°C for 16 h on 1% (w/v) agar plate of Luria-Bertani (LB) (Sambrook et al., 1989) containing 50 mgL^-1^ Kanamycin, 1.6 mgmL^-1^ X-Gal and 2.5 mmol IPTG to select the white colony of insert plasmid (so-called p35S-*ipt*). On the other hand, the recipient plasmid of p35S-*ipt* was modified using tissue-specific promoter, KNOX1 promoter cloned from *Elaeis guineensis*. The sequence of CaMV35S promoter region from p35S-*ipt* was removed using *Hind*III and *Bam*HI (Thermo Fisher Scientific, America) digestion and ligated with EgKNOX1 promoter driven a bacterial *ipt* gene (GenBank accession number: KJ634148.1), so-called pKNOX-ipt. The confirmation of insert *ipt* gene in both types of recombinant plasmids was investigated by PCR and sequencing analysis. The illustration of p35S-*ipt* and pKNOX-*ipt* were demonstrated in **Figure S1**.

The binary vector system was established in *Agrobacterium tumefaciens* strain EHA105 (Hood et al., 1993), which contained helper Ti-plasmid of *Agrobacterium* and Kanamycin resistant gene for bacterial selectable marker. The wild-host-range replicon plasmids (p35S-*ipt* or pKNOX-*ipt*) in *E. coli* were extracted by alkaline lysis method (Birnboim and Doly, 1979) and transformed into *A. tumefaciens* cells using electroporation system (Gene Pulser^®^ II, BIO-RAD). The *Agrobacterium* cells with recombinant plasmids were selected and multiplied by pour-plate technique on LB agar plate containing 50 mgL^-1^ Kanamycin and incubated at 28˚C for 2 days. The positive competent plasmids were confirmed by PCR analysis of *bar* and *ipt* genes, whereas the plasmids with different promoters of CaMV35S or KNOX1 were identified by PCR analysis of 35S and KNOX sequence (**Table S1**). The stock solution of *A. tumefaciens* with target competent plasmids were maintained according to the modified method suggested by Banyai et al., 2010.

### Plant materials

Sterilized seeds of *Peperomia pellucida* (L.) Kunth were collected from Bangkok, Thailand [13.7647355,100.523256,17] according to https://www.google.com/maps/place/Faculty+of+Science,+Mahidol+University+Phayathai/@13.7647355,100.523256,17z/data=!3m1!4b1!4m5!3m4!1s0x30e2994c6ef632db:0x2eed15a92965e95a!8m2!3d13.7647355!4d100.5254447. The seeds were surfaced sterilized with 10% Clorox^®^ solution (0.1% active ingredient of sodium hypochlorite) followed with 3 times washing by distilled water prior to germinate onto 0.1% (w/v) Gelrite^®^-solidified MS medium (Murashige and Skoog, 1962) containing 1% (w/v) sucrose. They were incubated at 25 ± 2°C, 60 ± 5% relative humidity and 16 h photoperiod with 60 ± 5 µmol.m^-2^. s^-1^ of photosynthetic photon flux density (PPFD). Node segments were excised from 7-day-old seedling of *P. pellucida* and used as donor explants for transformation.

The bialaphos sensitivity of *P*. *pellucida* was examined and further used as index to distinguish between the transformed plants and non-transformed escapes. Different concentrations (0, 0.5, 1.0, 1.5, 2.0, 2.5, 3, 3.5 or 4 mgL^-1^) of bialaphos (Meiji Seika Kaisha, Ltd., Tokyo, Japan) were supplemented into solidified MS medium containing 3% sucrose. After incubation in these medium compositions for 1 month, no survival was detected in medium containing bialaphos at ≥ 3 mgL^-1^. Therefore, bialaphos at 3 mgL^-1^ will be used in selection media of putative transformants.

### *Agrobacterium*-mediated transformation method and selection of putative transformants

A single colony of *A*. *tumefaciens* was selected from LB agar plate and transferred into 125 mL Erlenmeyer flask containing 30 mL of LB broth supplemented with 50 mgL^-1^ Kanamycin, the solution was incubated in rotary shaker (250 rpm) at 28˚C for 16 hrs. The bacterial pellet was collected by centrifugation at 1,500 rpm for 15 min and re-suspended in 30 mL of LB broth. The bacterial density was measured using spectrophotometric determination (GENESYS™ 10S UV-Vis Spectrophotometer) at the absorbance of 600 nm (OD600) for approximately 1.0, then centrifuged at 10,000 rpm (15 min). The bacterial pellets were re-suspended in MS liquid medium containing 100 µM acetosyringone (Sigma-Aldrich, UK) and used as cocultivated bacterial solution with node segments.

The node explants of *P. pellucida* were immersed into 30 mL of cocultivated solution in 125 mL Erlenmeyer flask. They were sonicated for 5 min in sonicator (Aczet Ultrasonic Cleaner CUB), followed with 15 min of vacuum infiltration using aspirator pump (Büchi^®^ Model B-169). The infected explants were placed on 0.1% (w/v) Gelrite^®^-solidified MS medium containing 100 mgL^-1^ acetosyringone and incubated at 25 ± 2°C under dark condition for 2-3 days. Shoot/root formation was observed after transferred the node segments to 0.1% (w/v) Gelrite^®^-solidified MS medium containing 100 mgL^-1^ Meropenem (Sigma-Aldrich, UK) as antibacterial agent to suppress and eliminate *A. tumefaciens*. The putative transformants were selected on MS medium containing 3 mgL^-1^ bialaphos (Meiji Seika Kaisha, Ltd., Tokyo, Japan) by subsequently subcultured every 30 days into same medium composition. The percentages of survival and plant regeneration on selectable media were observed (survival of transformation (%) = number of bialaphos-resistant plantlets regenerated in 3 mgL^-1^ bialaphos-containing media/total number of explant X 100). After three months of selection, the transformants were tested for positive PCR analysis of *bar* gene prior to confirm by Southern blot hybridization.

### Screening of putative transformants by PCR analysis of *ipt* gene

PCR analysis was used to rapidly screen putative transformants with positive *ipt* gene. The plant DNA was isolated from 200 mg fresh weight (FW) of *P. pellucida* leaves using the modified CTAB (cetylmethyl-ammonium bromide) method (Murray and Thompson, 1980). PCR analysis was performed using a programmable DNA thermal cycler (Perkin Elmer, USA). The reaction was operated in 25 μl final volume containing 1 μl DNA template with the following thermal cycling conditions: 34 cycles of 5 min at 95°C, 1 min at 55°C for *ipt* gene, and 1 min at 72°C. The 1.9 kb fragment of *ip*t was amplified with specific forward and reverse primers (**Table S1**) as follows: 5’ATGAGCCCAGAACGACGCCCG3’ and 5’TCAAATCTCGGTGACGGGCAGG3’. PCR products were electrophorized on 1.0% agarose gel at 100 volts and visualized under LED Transilluminator by Gel doc™ after staining with 0.1% ethidium bromide. The percentages of *ipt* -positive PCR were evaluated among different transgenic lines of generation T_0_, T_1_, T_2_ and T_3_ in order to examine the transformation evidence and further confirm by Southern blotting.

### Confirmation of *bar* and *ipt* genes in genomic DNA of transgenic *P*. *pellucida* by Southern blot hybridization

The transgenic plantlets of *P*. *pellucida* with *ipt*-positive PCR were further confirmed for the integration of *bar* and *ipt* gene using Southern blot analysis. The 200-µg aliquot of isolated DNA were digested by *Hind*III and *Xho*I (BioLab, New England), the restriction enzymes with single cutting site in T-DNA region at 37 ˚C for 20 h. (Ohara et al., 2000). The digested DNA was separated by electrophoresis in 0.8% (w/v) agarose gel and transferred to Hybond N^+®^ membrane (Amersham Biosciences, UK). The labeled *bar* and *ipt* probe were prepared according to the manufacturer’s instructions (Roche, Germany) and hybridized overnight on the membrane containing DNA fragment at 68°C. The signals were detected on membrane by using the NBT/BCIP ready-to-use tablet (Roche, Germany).

### Transcriptomic analysis of bacterial *ipt* gene and plant *ipt*3 gene

Leaves of wildtype and transgenic lines of *P. pellucida* were pre-cooled in liquid nitrogen and homogenized into fine powders in a BioPulverizer (BioSpec Products Inc., Bartlesville OK). The protocol for RNA extraction was modified from CTAB method. The sample powders were transferred to RNase-free microcentrifuge tubes filled with 600 µl pre-warmed CTAB extraction buffer and vortexed for 5 min at room temperature. The aliquot volume of chloroform-isoamyl alcohol (Fisher Scientific, Pittsburgh PA) at 24:1 was added, mixed well and centrifuged (15,000 rpm) for 5 min at 4°C. After centrifugation, the supernatant was re-extracted with an equal volume of chloroform-isoamyl alcohol, then mixed with an equal amount of isopropanol (IPA) and centrifuged (15,000 rpm) for 15 min at room temperature. The supernatant was removed, washed pellets in 1.0 ml of 75% ethanol and centrifuged at 15,000 rpm before dissolved pellets in 30 - 50 µL RNase I (Invitrogen, USA) to eradicate remaining DNA. The resulting RNA was stored at -80°C. The absorbance of RNA in RNase-free water was evaluated at 230, 260 and 280 nm using a NanoDrop ND-1000 spectrophotometer (Thermo Scientific, Wilmington DE). RNA yield was calculated based on the Beer-Lambert Law, according to which the yield in µg/mL is 40X the absorbance at 260 nm (A260). RNA purity was assessed by the ratios of A260/A280 and A260/A230. RNA integrity was evaluated by the ratio of 28S/18S ribosomal RNA (rRNA) and the RNA integrity number (RIN) using an Agilent 2100 BioAnalyzer (Agilent Technologies, Foster City CA). After the cDNA synthesis using the Primerscript RT Reagent Kit with gDNA Eraser (Invitrogen), qRT-PCR was performed using SYBR Premix Ex Tag (Invitrogen) and cDNA as the template following the manufacturer’s recommended protocol.

The bacterial *ipt* and plant endogenous *ipt*3 gene (Takei et al., 2001) were investigated (**Figure S3**). The specific forward and reverse primers of *ipt* gene (**Table S1**) were 5’-ATGAGCCCAGAACGACGCCCG-3’ and 5’-TCAAATCTCGGTGACGGGCAGG-3’. The primers of *ipt*3 gene were 5′-CATGGCGAATCTCTCCATTGA-3′ and 5′-AGTTGGAACCTCCAACGATGA-3′. The relative expressions of mRNA were evaluated by compare with Actin2 (*ACT2*) that used as the reference gene for normalization (forward primer: 5′-TTGTTTGTTTCATTTCCCTTTTTG-3′, reverse primer: 5′-GCAGACGTAAGTAAAAACCCAGAGA-3′). Accumulation levels of targeted transcripts were analyzed by real-time PCR method using ABIPRISM 7000 Sequence Detection System (Applied Biosystems) to monitor the amplification with SYBR-Green I dye (Applied Biosystems). Relative expressions of bacterial *ipt* and plant *ipt3* genes were determined according to the 2(-Delta Delta C(T) method (Livak and Schmittgen, 2001; Livak and Schmittgen, 2008). Mean CT values and standard deviations (SD) were used in the ΔΔCT calculations in 2^−ΔΔCT^ method to analyze the relative changes in gene expression from real-time quantitative PCR experiments by equation of ΔΔCT = ΔCT (a target sample) - ΔCT (a reference sample). The threshold cycles (Ct) of target genes were standardized to the Beta-actin Ct (ΔCt). The final result of this method was presented as the fold change of target gene expression in a target sample relative to a reference sample, normalized to a reference gene. The experiments were performed using five biological replications per treatment with three experimental replications of quantitative real-time PCR. Finally, the amplification product (10 µl) was analyzed and visualized by 1.0% gel electrophoresis using 1 kb DNA ladder (5 μL). The image was visualized under LED Transilluminator by Gel doc™ after staining with 0.1% ethidium bromide.

### Evaluation of morphological development and the phenotypic characteristics of transgenic *P. pellucida*

Growth and development of transgenic plants among different generations (T_0_, T_1_, T_2_, T_3_) and wildtype were evaluated at vegetative and reproductive stages. The shoot height (cm), number of shoots, number of leaves, leaf area (cm^2^) and characteristics of leaf and branching were observed during vegetative growth. At reproductive stage, the numbers and length (cm) of inflorescence, pollen viability, numbers of seed and seed size (μm) were examined. On the other hand, the period (days) presented in each step of growth and development were characterized among different transgenic lines in comparison with wildtype. The pollen viability was tested using safranin solution for staining according to method of Aldahadha et al. (2020). Safranin solution was prepared by dissolving 1 g of safranin in 40 mL of 95% alcohol and then adding 60 mL distilled water to make 100 mL stock solution. The pollens were immersed in staining solution (mixing solutions of 20 mL safranin with 40 mL glycerol and 20 mL distilled water) for 1 hour. The viable pollens presented as red staining were determined under light microscope. The percentage of viability was calculated from three experimental replications, one replication referred to one micro-slides that smeared with pollen grains (the density was adjusted with UV spectrophotometer at the absorbance of 450 nm for approximately 1.0). Pollen viability percentage was calculated using this equation: Pollen viability (%) = (Number of viable pollen grains/Number of incubated pollen grains) x 100. The pollen tube growth was tested by spreading the pollen grains onto germination slide containing Brewbaker and Kwack medium (Brewbaker and Kwack, 1964) and incubated under 25 ± 2 °C for 24 hours. The pollen tube growth was observed under light microscope using 3 different areas of hemocytometer (1 × 1 mm^2^). The germination percentages were calculated from total one hundred pollen grains and calculated using the equation: Pollen germination (%) = (Number of germinated pollen grains/Number of incubated pollen grains) x 100.

The surface features and structures of seed and shoot apical meristem were visualized by Scanning Electron Microscopy (SEM). The mature seeds of the wildtype and transgenic *P. pellucida* were removed from dry inflorescence. Seed coats were vapor fixed in OsO4 and sputter-coated with carbon and gold. Specimens were examined using a Scanning Electron Microscope (SEM; Hitachi SU-8010) at 10,000X magnification in high vacuum mode. For shoot apical meristem (SAM), the specimens were fixed in 3% glutaraldehyde in phosphate buffer, pH 7.2 for 24 h. After fixation, the samples were rinsed with phosphate buffer, dehydrated in ethanol, and stored at 4°C. Tissues were subsequently critical point dried using liquid carbon dioxide. Dried samples were covered with a carbon-gold palladium mixture and examined using a Hitachi SU-8010 Scanning Electron Microscope operated at 10 kV. The images obtained by phase contrast microscopy visualization demonstrated the characterization of plant tissues and sections.

### Photosynthetic pigment content

Chlorophyll plays a critical role in plant growth and contribute greatly to the phenotypic expression. The variation of chlorophyll also related to cytokinin biosynthesis pathway through the MEP pathway. The chlorophyll measurement was modified from Alan’s method (Alan, 1994) as follows: 0.5 g of fresh leaves were homogenized in 5 ml of 80% acetone and kept at 4°C for 48 h. The extracts were then centrifuged at 10,000g for 2 min at 4°C and made up to 1 ml with 80% acetone. The supernatant was used for spectrophotometric determination of chlorophyll a (Chl_a_) and chlorophyll b (Chl_b_) at absorbances of 663 and 645 nm, respectively. Total chlorophyll was calculated by the standard equations in units of milligrams per milliliter (mg/ml): Chl_a_ = 12.7A663 – 2.69A645, Chl_b_ = 22.9A645 – 4.68A663. Results were expressed as milligrams of chlorophyll per gram dry weight. Total Chlorophyll (mg/mL) = Chl_a_ + Chl_b_. Total Chlorophyll (mg) in original tissue sample = Total Chlorophyll (mg/mL) x final volume (mL).

### Cytokinin analysis in *P. pellucida* leaves

Cytokinin extraction was followed the protocol as described previously (He et al., 2005). About 0.2 g fresh leaves were extracted and homogenized in 2 ml of 80% methanol containing 40 mg L^-1^ butylated hydroxytoluence and stored at –20°C for 48 h. After centrifugation at 10,000 rpm for 15 min, sediments were resuspended in 1 ml 80% methanol at -20°C for 16 h. Afterwards, samples were evaporated under vacuum to remove the organic solvent and dissolved in 2.0 ml of Tris-biphosphate (TBS) buffer to determine ZR (Zis-zeatin), and iPA (isopentenyladenosine). The amounts of endogenous cytokinin, ZR and iPA were determined by ELISA using monoclonal antibodies by following the protocol provided by the manufacturer (Phytodetek, Agdia, Elkhart, IN, USA). The absorbance was recorded at 405 nm and calculated using microplate reader (Tecan Spark™ 10M) as described in Weiler et al. (1982).

### Statistical analysis

All experiments were set up in completely randomized design. Data were analyzed by one-way ANOVA using IBM Statistical Package for the Social Science (SPSS) version 18 (Software License Download@ Mahidol) to determine the significant differences between means ( ± SE) using Duncan’s Multiple Range Test (DMRT). The standard error (SE) was determined in each experiment.

## Results

### Roles of pKNOX1-driven bacterial *ipt* on stability and reproducibility of transgene inheritance in *P. pellucida*

After pCaMV35S-*ipt* and pKNOX1-*ipt* gene fragments with *bar* gene as selectable marker (**Figure S1**) were transformed to *P. pellucida* by *Agrobacterium*-mediated transformation, the 30 day-transformants were selected on selectable medium containing 3 mgL^-1^ bialaphos. Four lines of *ipt* transgenic plants were confirmed the transgene insertion by PCR and Southern blot analysis (data not shown), c-11 and c-12 for plants with CaMV35S and k-14, k-20 with KNOX1 promoter. The genetic inheritance of *ipt* genes and the number of survived transgenic plants were continuously tracked from T_0_ to T_3_ generations (**Table 1**). The result showed that transgenic plants with KNOX1 promoters produced fewer seeds than plants with CaMV35S but had higher rates of survival and success in gene transfer to the next generation in terms of percentage of survival plants and number of PCR positive plantlets. The percentage of survivals in two lines of transgenic plants with KNOX1 promoter (1.25%) showed 2.2 folds more than obtained from the transgenic with p35S (0.58%). Quantitative examination of *ipt*-positive gene expression in T_3_ generation of transformants with pKNOX1 were 100% (line k-14) and 50% (line k-20), while 33.3% was observed in transgenic line c-11 with p35S. Most of seed-derived transgenic *P. pellucida* with CaMV35S promoter provided lower reproductive fitness. The seeds of c-12 at T_1_ generation containing two copy numbers of *ipt* genes inserted into the plant genome, as confirmed by Southern blot analysis (**Figure S2**), could not survive, and produce progeny in further T_2_ and T_3_ generations. This was caused by overexpression of the *ipt* gene under the control of the constitutive promoter, pCaMV35S, which affected seed germination and developmental processes. High number of *ipt* transplants in T_3_ and stable genetic transformation events in progeny using pKNOX1 studied here were efficient for breeding programs. pKNOX1 acted as a regulatory specific promoter driving the expression of the *ipt* gene to activate the functional transgene for stable inheritance to three generations in *P. pellucida*, the particular plant model.

**Table 1:** The genetic inheritance of transgenes (*ipt*) in T_1_, T_2_ and T_3_ generations of transgenic *P. pellucida* plants obtained from two different promoters as c-11 and c-12 (driven by CaMV35S promoter), k-14 and k-20 (driven by KNOX1 promoter).

Transgenic lines	Transgenic seeds	Tested seedlings	Survival(%) ^1/^	Plants (%) with PCR positive *ipt*	Copy number of *ipt* gene ^2/^
**CaMV35S**	T_1_	398	14.32	1.75	1
** c-11**	T_2_	331	2.42	12.50	
	T_3_	116	2.59	33.33	
** c-12**	T_1_	117	0.00	0.00	2
	T_2_	0	0.00	0.00	
	T_3_	0	0.00	0.00	
**KNOX1**	T_1_	228	23.68	1.85	1
** k-14**	T_2_	205	12.20	16.00	
	T_3_	74	4.05	100.00	
** k-20**	T_1_	172	16.28	3.57	1
	T_2_	144	9.03	23.08	
	T_3_	51	3.92	50.00	

^1/^ Percentage of bialaphos-resistant plantlets after 30 days of germination on 0.2% Gelrite^®^-solidified MS medium containing 1% (w/v) sucrose and 3 mgL^-1^ bialaphos (Meiji Seika, Tokyo, Japan).

^2/^ Copy number of *ipt* in transgenic *P. pellucida* was confirmed by Southern blot hybridization. Investigation of transgene inheritance in each generation was evaluated from seed-derived transplants in terms of survival (%), PCR positive plantlets (%) and copy number of tested transplants.

### Morphological and reproductive characteristics of *P. pellucida* containing pKNOX1-driven bacterial cytokinin biosynthesis gene

*In vitro* shoots and leaves formations of transgenic *P. pellucida* were observed after 4 weeks of *Agrobacterium*-mediated transfer T-DNA containing the isopentenyltransferase (*ipt*) gene responsible for cytokinin biosynthesis and the *bar* gene, a selectable marker gene that encoded phosphinothricin acetyl transferase (PAT), driven by CaMV35S as constitutive promoter and KNOX1 as tissue-specific promoter, respectively. Regenerated transgenic plants were screened by cultured on MS medium with herbicide bialaphos used as the selectable agent at concentrations of 2 mgL^-1^ and transplanted to vermiculite-supporting media. 60-day transgenic plants showed different characteristic features compared to wildtype plants.

**Figure 1:**
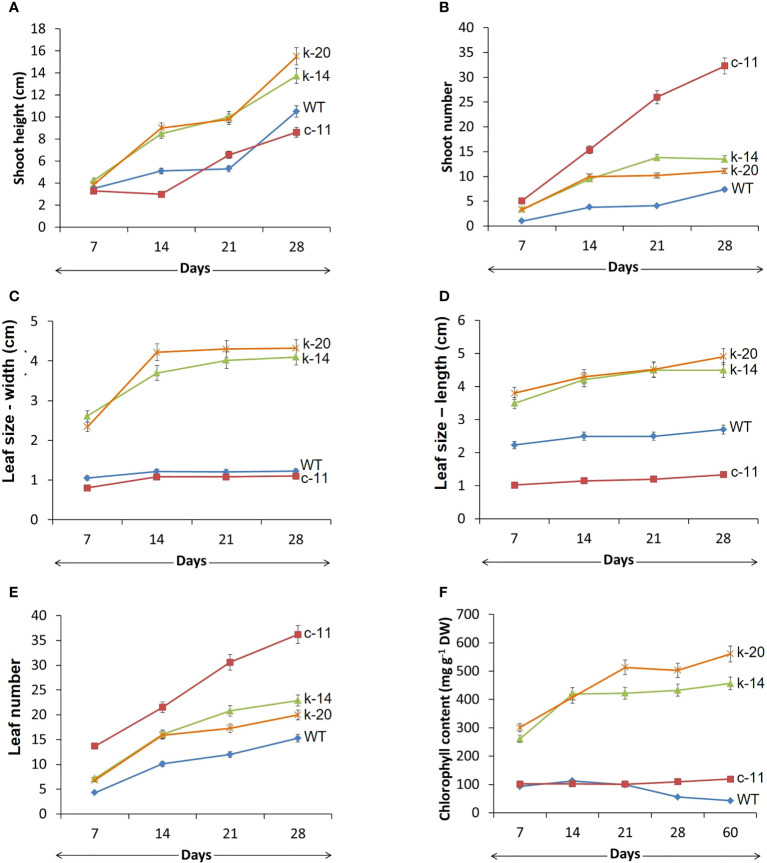
Morphological growth of wildtype (WT,
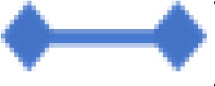
) and transgenic *P. pellucida*; c-11 (
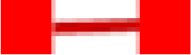
) driven by CaMV35S promoter, and k-14 (
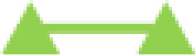
) and k-20 (
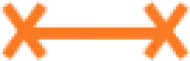
) driven by KNOX1 promoter. Comparison of shoot height **(A)**, number of shoots **(B)**, leaf size in y axis of width **(C)** and length **(D)**, number of leaves **(E)** and chlorophyll content **(F)** of seed-derived plantlets of WT and T1-transgenic *P. pellucida* after 7, 14, 21 and 28 days of germination in a controlled growth cabinet. Data (mean ± SE) were analyzed from 20 biological replications per treatment and showed highly significant difference at p ≤ 0.01. Line c-12 obtained from p35S transgenic plants remained only two biological replications, which could not perform for statistical analysis.

**Figure 2:**
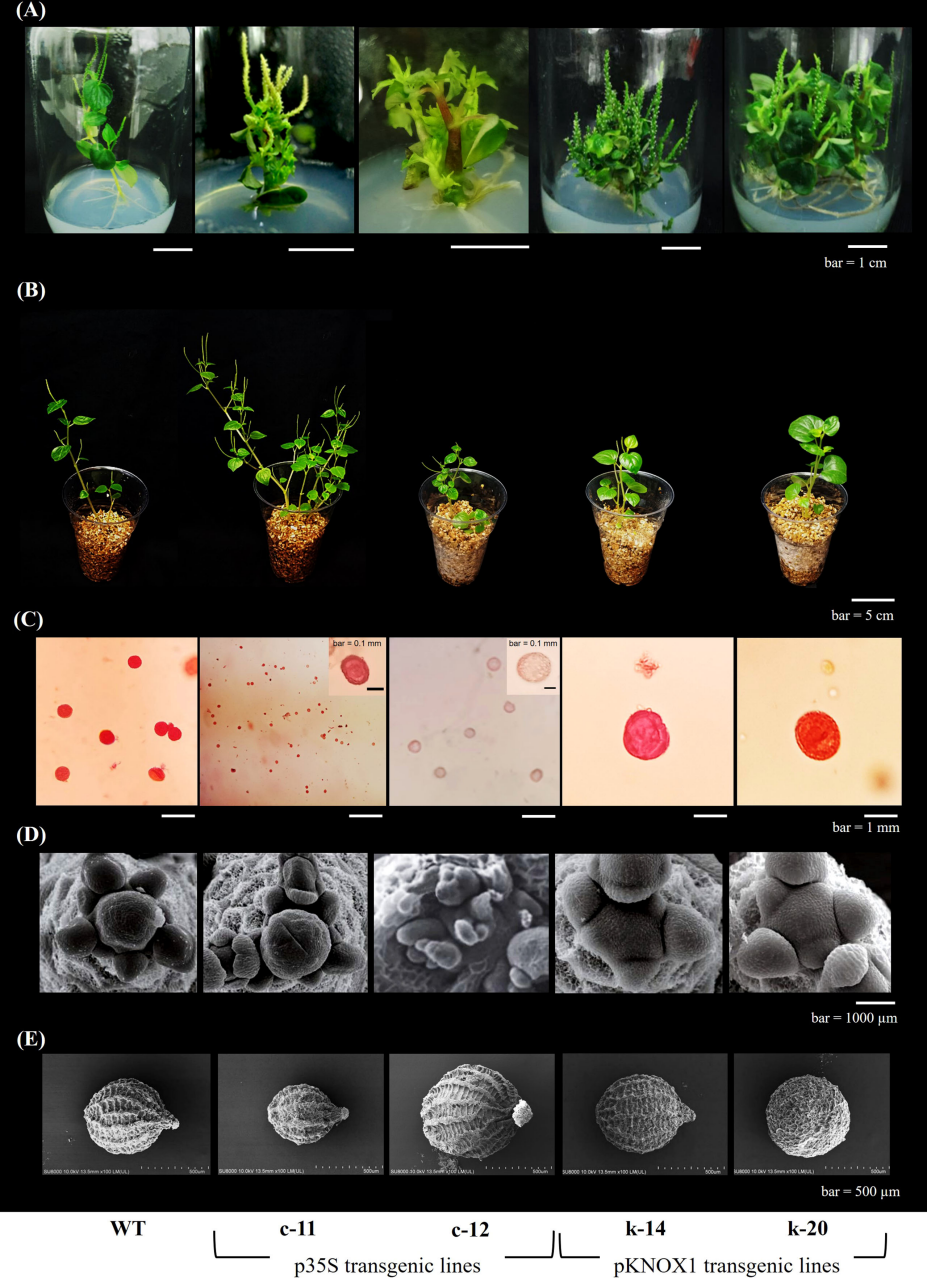
Phenotypic characters of *P. pellucida* reveal for *in vitro* plantlets **(A)**, morphological growth of transplants at 30 days of transplantation **(B)**, pollen size and viability stained with safranin **(C)**, scanning electron microscope (SEM) of shoot apical meristem **(D)** and SEM of seed **(E)**. The T_1_-transgenic lines obtained from different promoters as p35S transformant (c-11) and pKNOX1 transformants (k-14 and k-20) were compared with wildtype (WT).

**Figure 3:**
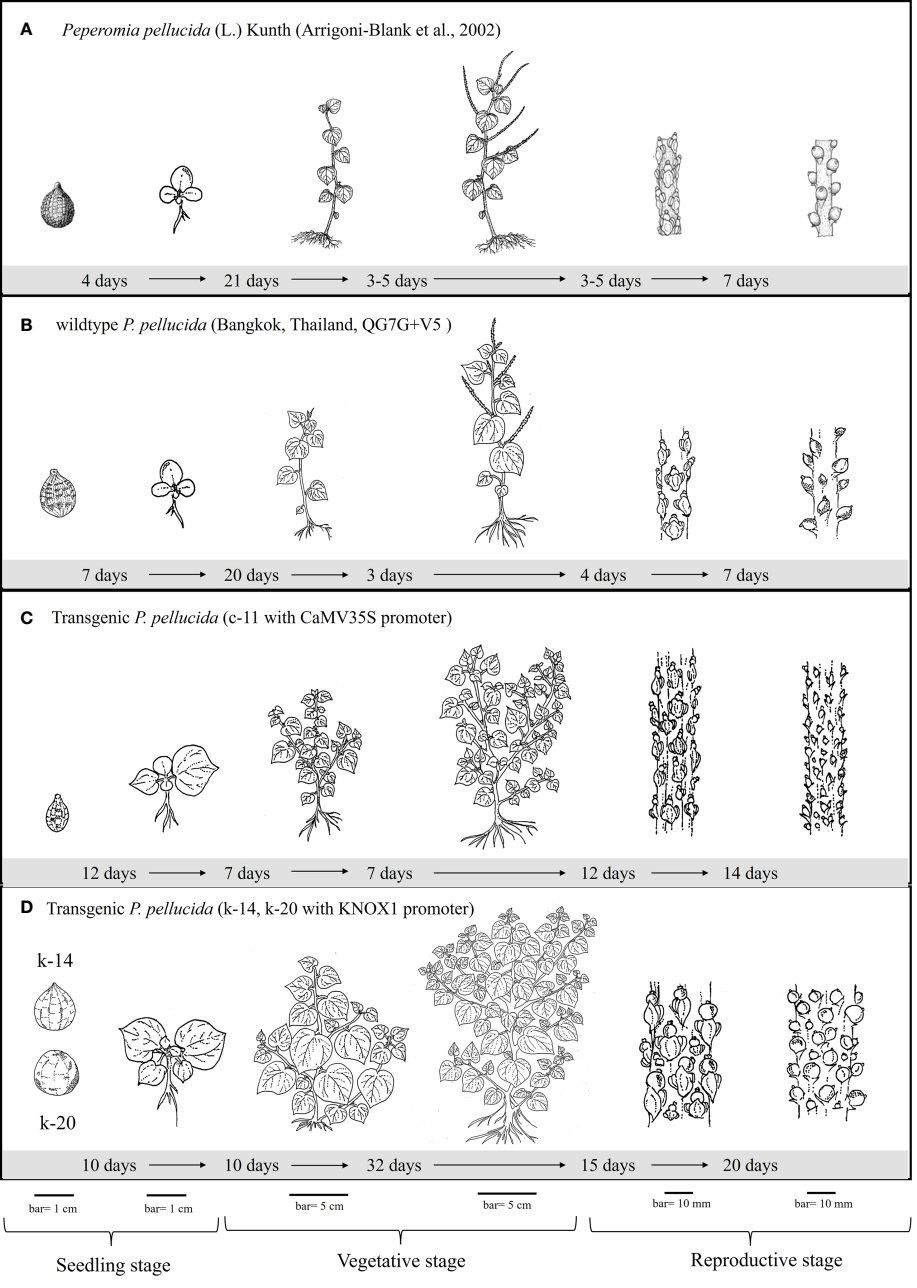
Illustration of growth and developmental performances demonstrate the characteristics of seed (bar = 1 cm), seedling (bar = 1 cm), branching shoot with leave (bar = 5 cm), inflorescence and seed set (bar = 10 mm) of wildtype **(B)** and transgenic *P. pellucida* plants; line c-11 obtained from CaMV35S promoter **(C)** in comparison with those transgenic lines k-14 and k-20 obtained from KNOX1 promoter **(D)**. The growth and developmental cycle were compared according to the reference of specimens collected from gardens and fields **(A)** reported by Arrigoni-Blank et al. (2002). The location of *P. pellucida* wildtype presents in **(B)** is referred to Faculty of Science, Mahidol University [13.7647355,100.523256].

In this experiment, *in vitro* explants with CaMV35S promoter also showed abnormalities in leaves and inflorescence of all clones (**Figure 1**, **Figures 2A–C**, **Figure 3C**, **Table 2**). One of the most characteristic features of the *ipt* transgenic plant with CaMV35S promoter was loss of apical dominance, producing significantly more branches than wildtype at vegetative stage. The extreme branching phenotype was formed due to the continuous formation of higher order branches by overexpression of *ipt* gene releasing dormant axillary buds. The pCaMV35S-*ipt* leave were significantly smaller and the inflorescence stems were slender than the wildtype (**Figures 2A, B**). After 30 days of transplantation to vermiculite, T_1_ plants with KNOX1 promoter at the vegetative stage showed significantly greater main stem height and leaf size than WT and transgenic plants with CaMV35S but fewer numbers of shoots and leaves were recorded in CaMV35S transgenic plants. Leaves of CaMV35S transgenic plants were smallest compared to other experimental sets. Leaf size of CaMV35S plants was 13-15 folds and approximately 2 times smaller than plants with KNOX1 and wildtype, respectively. Size and characteristics of shoot apical tissue (SAM) were imaged by scanning electron microscopy (SEM). Both apical initial tissue and the central mother cell zone responsible for cell division, proliferation and expression domains of genes related to cell differentiation, and peripheral zones involved in leaf primordia formation were all larger and more mature compared with plants with CaMV35S and WT (**Figure 2C**), resulting in development promotion of leaves and shoots.

**Table 2:** The reproductive characteristics (flowering date, inflorescence number, inflorescence length, pollen viability, pollen tube germination, seed-setting date, seed number and seed size) of wildtype (WT) and transgenic lines of *P. pellucida* (c-11, k-14 and k-20) after 60 days of transplantation.

Developmental parameters	Wildtype (WT)	p35S transgenic	pKNOX1 transgenic		Significant level
		c-11	k-14	k-20	
Flowering date (day after germination)	29.8 ± 0.6 b	25.1 ± 0.6 c	52.0 ± 0.5 a	50.9 ± 0.8 a	**
Inflorescence number	13.3 ± 1.4 c	42.2 ± 4.0 a	22.2 ± 0.8 b	23.0 ± 0.6 b	**
Inflorescence length (cm)	4.4 ± 0.2 c	5.4 ± 0.1 b	6.3 ± 0.2 a	6.8 ± 0.1 a	**
Pollen viability (%)	85.2 ± 0.4 a	23.8 ± 0.6 b	80.4 ± 0.2 a	81.7 ± 0.2 a	**
Pollen germination (%)	77.5 ± 0.6 a	10.3 ± 0.6 b	71.0 ± 0.6 a	69.6 ± 0.6 a	**
Seed-setting date (day after germination)	33.7 ± 0.6 b	38.4 ± 0.9 b	67.0 ± 0.9 a	66.9 ± 1.0 a	**
Seed number	131.0 ± 6.5 d	953.6 ± 8.3 a	237.2 ± 7.6 c	286.3 ± 5.6 b	**
Seed size (μm)	432.5 ± 0.5 b	250.4 ± 1.0 c	500.0 ± 1.3 a	512.3 ± 1.1 a	**

Different letters within row indicate for highly significant differences of means (± SE) at p ≤0.01 analyzed by Duncan’s Multiple Range Test (DMRT) using 20 biological replications of each *P. pellucida* line (1 pot plant/1 replication). P values of statistical significance less than 0.01 as statistically highly significance are summarized with two asterisks **.

Chlorophyll content measured from the leaves of KNOX1plants was significantly higher than in the other experiments, generating more photosynthetic compounds (**Figure 1F**, **Table 2**). Sixty days after transplanting the plants produced new shoots and leaves continually without flowering and fruiting. Chlorophyll content in CaMV35S transgenic plants was relatively stable from measurements taken 30 days after transplantation, with thin, small mature leaves leading to small amounts of chlorophyll. Pale leaves of 30-day-old pCaMV35S-*ipt* plants were shown observed in **Figure 2B**. The pCaMV35S-*ipt* plants maintained 10-13-fold higher levels of total chlorophyll content at 60 days after transplantation without reduction or degradation of chlorophyll depending on age, while WT plants showed a gradual decrease in chlorophyll content at 21 days after transplantation. The pKNOX1-*ipt* transgenic plants maintained chlorophyll contents about 4-5 folds extending the developmental cycle to 12.4 weeks, which was 2 folds more than wildtype (5.8 weeks) and p35S-transformants (7.4 weeks).

Size and morphology of transgenic *P. pellucida* seeds were significantly different compared with WT (**Figure 2D**). CaMV35S plants had smaller seeds than the others, while seeds from plants containing pKNOX1 were large, with a smooth textured seed coat surface and spherical in shape. These seeds could also survive and maintain genes inheritance to the next generation. The reproductive stage of transgenic lines propagated to T_1_, T_2_ and T_3_ generations were showed in **Table 2**. The reproductive stage of T_1_ plants with different promoters showed diverse characteristics. Numbers of inflorescences and seeds of KNOX1 plants were lower than plants with pCaMV35S but inflorescence length and seed size were significantly greater. Interestingly, the flowering and fruiting initiation dates of plants with KNOX1were twice as long as CaMV35S plants, indicating that the KNOX1 promoter maintained vegetative growth and leaf production while extending harvesting time and increasing leaf biomass. An overview of the phenotypic cycle of transgenic plants with different promoters compared with wildtype plants from seedling to vegetative and reproductive stages is shown in **Figure 3**. The periods of each stage differed from the normal life cycle of *P. pellucida* grown in field environments (Arrigoni-Blank et al., 2002), especially vegetative stage of pKNOX1-*ipt* plants that provided 4.57 and 10.67 longer periods than pCaMV35S-*ipt* and WT plants, respectively. Transgenic *P. pellucida* under controllable expression of pKNOX1 showed increased leaf and seed size with a high percentage of fertile seed (**Figure 2E**), whereas transgenic plants with p35S showed phenotypic features of bushy and small leaves, sterile pollens, and lower reproductive fitness. The pollen size and viability stained with safranin were showed in Fig 2. The pollen of pCaMV35S-*ipt*, line c-12 showed no pollen viability so there was no its progeny in T2 and T3 as shown in **Table 1**. Although pCaMV35S-ipt, line c-11 performed their reproductive fitness ability to T_3_, the low percentage of pollen viability and pollen germination were showed in **Table 2** as about 4 and 7 folds in comparison with pKNOX1-*ipt* plants.

### Overexpression of *ipt3*, a plant cytokinin biosynthesis gene was found more than the bacterial *ipt* gene in pKNOX1: *ipt*-transgenic *P. pellucida* relating to cytokinin content

Four plant lines of transgenic *P. pellucida* generation T_1_ in *in vitro* condition resulting from independent transformation events were confirmed by PCR amplification and Southern blotting analysis (data not shown). After gene expression, the transformed plants were activated by bialaphos for 48 h and relative expression levels of the *ipt* gene were analyzed.

**Figure 4:**
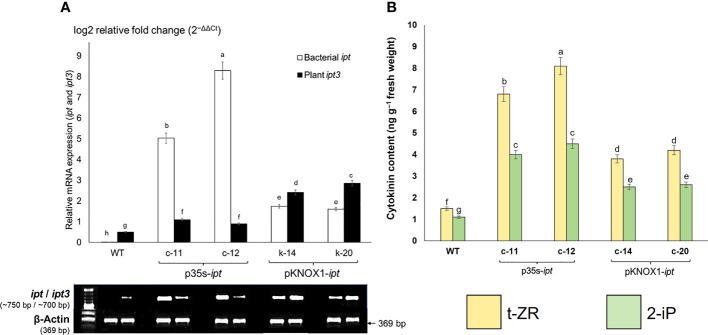
Transcriptomic expressions of cytokinin biosynthetic genes **(A)** comprise with bacterial *ipt* (GenBank accession number: KJ634148) and *ipt3* (isopentenyltransferase; GenBank accession number: TR435612); and cytokinin contents **(B)**. Quantitative RT-PCR showing relative mRNA expressions determined using the 2^(−ΔΔCt)^ method, threshold cycles (Ct) for target genes are standardized to the Beta-actin Ct (ΔCt). Transcriptomic expressions of bacterial *ipt* (white bar) and plant *ipt3* (black bar) in wildtype (WT) and transgenic *P. pellucida* were compared. Four transgenic lines derived from different promoters; pCaMV35S (c-11, c-12 lines) and pKNOX1 (k-14 and k-20 lines). Gel electrophoresis of mRNA levels were determined using Beta-actin as reference and 1 kb molecular marker **(A)**. The amounts of endogenous cytokinin **(B)** were determined by ELISA using monoclonal antibodies by following the protocol provided by the manufacturer (Phytodetek, Agdia, Elkhart, IN, USA). The levels of trans-zeatin riboside (t-ZR) and isopentenyladenine (2-iP) were examined in WT and four lines of transgenic *P. pellucida*. Different letters of **(A)** and **(B)** indicate highly significant differences of means ( ± SE) analyzed by using Duncan’s Multiple Range Test (DMRT) at *p* ≤ 0.01. The experiment was performed using five biological replications per treatment and three experimental replications.

The bacterial *ipt* gene, driven by different promoters and inserted into the plant genome, affected the expression of *ipt3*, a common gene involved in endogenous cytokinin biosynthesis in plants. In this study, two lines of transgenic *P. pellucida* plants containing the *ipt* gene driven by CaMV35S promoter (c-11, c-12), respectively showed relative expression 2-3 times higher than plants with KNOX1 promoter (k-14, k-20), respectively, while the bacterial *ipt* gene was not expressed in WT. By contrast, the expression levels of *ipt3* spontaneously generated in plants with KNOX1 promoter significantly increased compared with the others (**Figure 4**). The endogenous cytokinin biosynthesis gene (*ipt3*) that was significantly upregulated (2-3 folds higher) and the overall relative mRNA expression of bacterial *ipt* gene in pKNOX1-*ipt* transformants regulated to the overproducing of total cytokinin contents (t-ZR and 2-iP) detected in p35S-transformants as shown in Fig 4. In this experiment, the overall relative mRNA expression of bacterial *ipt* gene and high cytokinin content detected in p35S-*ipt* plants caused the tumor formation and morphological abnormalities in transgenic plants (**Figures 1A, B**; **Tables 1**, **2**).

## Discussion

Introducing a transgene into the plant genome by *Agrobacterium*-mediated transformation usually produces transgenic plants with low copy number of the target gene. This transgene inheritance is transmitted to progeny depending on the site of transgene integration that determines its stability (Filipecki and Malepszy, 2006). If integration and gene expression occur in a transcriptionally active area, the influenced characteristic features provide more functions. Patterns and levels of transgene expression depend on the particular gene, the specific gene construct, the regulatory specific promoter driving expression and the proper recipient plant that has a good buffering capacity for inserted genes inherited in the three earlier generations (T_1_, T_2_ and T_3_) or least T_1_ generation as a dominant trait (Yu et al., 2014). Lazzeri et al. (2003) presented evidence showing correlation between transgene insertion number and expression level. The presence of multiple transgene copies has also been implicated in transgene silencing and limited levels of gene expression, resulting in loss of progeny over three generations of transgenic lines. Production of lines with a single transgene copy or low copy numbers was suggested to be more stable, with reduced transgene silencing. In the recent studies, KNOX1 can be applied as a specific promoter regulating the expression of the *ipt* gene for stable inheritance without risking the escape of transgenes to three generations in *P. pellucida*.

Normally, the cytokinin biosynthesis key step enzyme, isopentenyltransferase, encodes *ipt* family genes in the root apical meristem and transfers them to the shoot apical meristem area at the apex. The enzyme condenses isopentenyl pyrophosphate (IPP) and dimethylallyl-pyrophosphate (DMAPP) to produce isopentenyl-AMP, which is later converted to cytokinin *via* the mevalonate (MVA) pathway and functions in the development of apical meristem to shoot, leaf and flowering buds in combination with other plant hormones. Therefore, apical meristematic zones are important for cytokinin biosynthesis and function. Knotted-like homeobox (KNOX) genes encode homeodomain containing transcription factors that modulate developmental processes and hormone homeostasis in plants, especially Class I KNOX genes in most monocots that are expressed only in the shoot apical meristems (Tsuda and Hake, 2015) to function in the maintenance of meristematic cells and development of plant meristem involved in the morphogenesis of lateral organs, the diversity of leaf morphology and inflorescence development (Hake et al., 2004). The expression profiles revealed a tissue-specific expression of Class I KNOX genes with the specific functions of cis-element in shoot apical meristem where the pluripotent stem cells were generated (Zhang et al., 2022) SAM-specific expressed KNOX1 promoter in this study was also obtained from *Elaeis guineensis*, a represented monocotyledonous plant.

In previous studies (Merewitz et al., 2011 and Merewitz et al., 2016) the overexpression of bacterial *ipt* driven by inducible synthetic promoters associated with increases of IPT activity and endogenous cytokinin level in tobacco that may regulate other functional proteins involved in reducing stress penalties and inducing targeting desirable phenotypes. Sa et al. (2001) also found that using different promoters to control the expression of the same gene influenced regulatory gene expression and RNA splicing in plants. CaMV35S, a constitutive promoter, regulated gene expression in all tissues throughout the whole plant, leading to overexpression of the inserted *ipt* gene that interfered with the transcriptional expression of other related genes, including *ipt3*, involved in endogenous cytokinin biosynthesis. Energy usage in response to introducing foreign genes into the plant genome also disrupt or delay plant growth and developmental processes. KNOX1, a tissue-specific promoter, regulated specific gene expression at shoot apical meristem (SAM), which contained young thin-walled cells ready to differentiate into plant organs as development and primary growth. The SAM-specific promoter directly imported genes to the target area responsible for leaf and shoot formation without energy consumption and controlled the balance of expression levels of *ipt* genes in this region (Zhang et al., 2021). The expression of *ipt* genes was regulated by suitable promoters, while active localization of target functional genes was important for plant growth, development, and morphological patterns. In this study, chlorophyll content of pKNOX1-*ipt* plants was significantly higher than in the other experiments and maintained the high levels to 60 days after transplantation without reduction or degradation of chlorophyll. Sa et al. (2001) determined that increased cytokinin in the biosynthetic system was associated with variations of other related compounds in the terpenoid biosynthesis pathway, including chlorophyll because its precursors, IPP and DMAPP, were also present in the mevalonate (MVA) pathway and the non-mevalonate pathway (MEP) as well as cytokinin. pKNOX1-transgenic plants also maintained chlorophyll contents to 60 days and delay senescence resulting in prolong the developmental cycle in vegetative stage. Although the life cycle in pKNOX1-*ipt* transgenic plant was extended, it performs prominent performances to increase leave biomass with stable transgene segregation. Generally, this plant will produce seeds and terminate within 5.3-5.8 weeks. After seed setting, leaf numbers were reduced and found rapid chlorophyll degradation referred to the evidence of leaf senescence. The pKNOX1-transgenic plants showed more branches, bigger leaves, and delayed leaf senescence to about 8 weeks, which provide superior features of longer vegetative stage. Peperomia leave is a major part of important biomass that contains several active compounds. Therefore, longer period to harvest leaves provide more benefit to produce more raw materials. Similarly, the overexpression of *ipt* induced higher chlorophyll contents, and delay in leaf senescence in canola. The *ipt*-transgenic plants were stay-green and improved leaf development under abiotic stress (Kant et al., 2015). The significant roles of tissue-specific promoters in regulating the expression of target gene have been previously reported in supporting our results, functional expression of *ipt* gene was controlled by senescence-specific SAG12 promoter that allowed the localization of elevating cytokinin contents and retained the chlorophyll contents at lower leaves, which were found to be highly effective in controlling *ipt* expression and had effect to delay leaf senescence and delay in flowering in lettuce (McCabe et al., 2001). The efficacy of the tissue specific promoter SAG12 in which cytokinin genes are expressed in leaves and has effects to delay leaf aging is not only apply in leafy vegetable but also reported in monocotyledonous plant as wheat (Sýkorová et al., 2008) and rice (Devi et al., 2012). The transformation efficiency of transgenic japonica rice under controlled of pSAG12 performed better functions of cytokinin over a constitutive promoter. In our present research, in addition that could be able to extend the vegetative period of leaf production by delay leave senescence in *Peperomia* transgenic plants, we also manifest here that the use of a SAM-tissue specific promoter resulted in 50% (k-20) to 100% (k-14) of T_3_ segregation through active reproductive organs than those of c-11 that controlled constitutive p35S (33.3%).KNOX1 protein also have function to modulate the abundance of gibberellin and cytokinin. The cytokinin levels were increased by activating the transcription of cytokinin biosynthetic gene Isopentenyl transferase3 (*ipt*3). The higher expression of cytokinin in transgenic plants activated cell division and maintain meristematic cell in the SAM, in order to influence the balance between differentiation and cell division in SAM (Jasinski et al., 2005; Yanai et al., 2005). The KNOX1 specific promoter in this study controlled the balance of expression proportions of spontaneously generated *ipt*3 Accumulation of the AtIPT3 transcript is most commonly detected in all organs but more abundant in photosynthetic organs, especially in shoot (Kakimoto, 2001). genes in plants and regulated the expression of bacterial *ipt* to the correct area. Therefore, transgenic plants showed reduced abnormalities such as stunted stems, small, curled leaves, and inability for seed propagation in tobacco caused by overexpression of the *ipt* gene (Van et al., 1993).

In conclusion, *KNOX1* as SAM-specific promoter induced expression of the *ipt* gene directly to shoot apical meristematic cells, the target area, resulting in high *Agrobacterium*-mediated transformation efficiency of transgenic plants, increase of the endogenous cytokinin biosynthesis gene and also activation of the expression of cytokinin activities to regulate growth and development and phenotypic cycles of transgenic plants. *Peperomia pellucida* (L.) Kunth can act as an alternative plant model system for further development of morphology and biochemical characteristics.

## Conclusion

In the current study, KNOX1 as SAM-specific promoter induced stable genetic inheritance of bacterial *ipt* gene that encodes isopentenyltransferase regulating cytokinin biosynthesis directly to particular tissue (shoot apical meristem) in *Peperomia pellucida* (L.) Kunth without compromising growth and yield. We have conducted a study to examine two keys’ elements relevant to the dynamic expression of *ipt* by first designing KNOX1 as a tissue-specific promoter for the required levels of gene expression in particular target area, that can minimize host genomic stress resulting in high *Agrobacterium*-mediated transformation efficiency of transgenic plants. Transgenic *P. pellucida* with KNOX1 promoter induced increasing expressions of endogenous plant isopentenyl transferase (*ipt3*) gene for 2-3 folds more than CaMV35S-transgenic plants, and 6 folds more than wildtype. The increasing levels of cytokinin biosynthesis in particular tissue of transgenic plants as a result of KNOX1 promoter have effects on phenotypic features of leave, branching, fruit set with fertile seeds. Moreover, these transgenics showed delayed reproductive period 2-3 folds more than wildtype and CaMV35S-transgenic plants. Secondly, using *P. pellucida*, a medicinal plant as an attractive model plant due to its short developmental cycle supported expedient tracking the stability of transgene inheritance in the transgenic progeny from T_1_ to T_3_. This plant can be further used to evaluate both yield potential and omics studies for future agriculture.

## Data availability statement

The datasets presented in this study can be found in online repositories. The names of the repository/repositories and accession number(s) can be found below: https://www.ncbi.nlm.nih.gov/nuccore/KJ634148; https://www.ncbi.nlm.nih.gov/protein/AIC75601.

## Author contributions

PW has mainly undertaken the research work, analyzed the data and written the manuscript. RSG has partly helped for manuscript revision and prepared for references used in responses to reviewers. The corresponding author, KS has been responsible as a principal investigator of the research project to provide idea, data interpretation, manuscript correction, proof reading and actions of whole correspondence during the paper submission. All authors contributed to the article and approved the submitted version for publication.

## Funding

The authors would like to thank the Royal Development and Promotion of Science and Technology Talents Project (DPST) scholarship to PW. This research (contract no. C10F640135) is granted by the Office of National Higher Education Science Research and Innovation Policy Council by the “Program Management Unit for National Competitiveness Enhancement (PMU-C)” to KS (PI) and the co-funding resources of AgrowLab Co., Ltd. Thanks are also due to the Central Instrument Facility (CIF), Faculty of Science, Mahidol University for experimental facilities supported.

## Conflict of interest

The authors declare that the research was conducted in the absence of any commercial or financial relationships that could be construed as a potential conflict of interest.

## Publisher’s note

All claims expressed in this article are solely those of the authors and do not necessarily represent those of their affiliated organizations, or those of the publisher, the editors and the reviewers. Any product that may be evaluated in this article, or claim that may be made by its manufacturer, is not guaranteed or endorsed by the publisher.

## Supplementary material

Supplementary Figure 1 Schematic diagrams of expression vectors under the control of CaMV 35S **(A)** and *KNOX*1 **(B)** promoter.

Supplementary Figure 2 Identification of representative of transgenic *P*. *pellucida* plant lines carrying integrated T-DNA resulted by PCR and Southern blotting analysis digested with *Bam*HI and *Xho*I. Molecular markers are indicated on the left. Lane M, molecular size marker 1 kb; Lane 1, transgenic control plant without *ipt* gene (P3300); Lanes 2–3, transgenic *ipt* plants with KNOX1 promoter (k-14, k-20); Lanes 4–6, transgenic *ipt* plants with 35S promoter (c-11, c-12, c-15); Lane 7, wildtype plant.

Supplementary Figure 3 Nucleotide sequence and conserved region of ***IPT*** and ***IPT3*** gene.

Supplementary Table 1 List of primer sequences used for PCR and qPCR analysis.
