# Supplementary figures and images for: Stable and reproducible expression of bacterial ipt gene under the control of SAM-specific promoter (pKNOX1) with interference of developmental patterns in transgenic Peperomia pellucida plants
Source: Front Plant Sci. 2022 Sep 27;13:984716. doi: 10.3389/fpls.2022.984716 (PMC9551203; doi:10.3389/fpls.2022.984716)

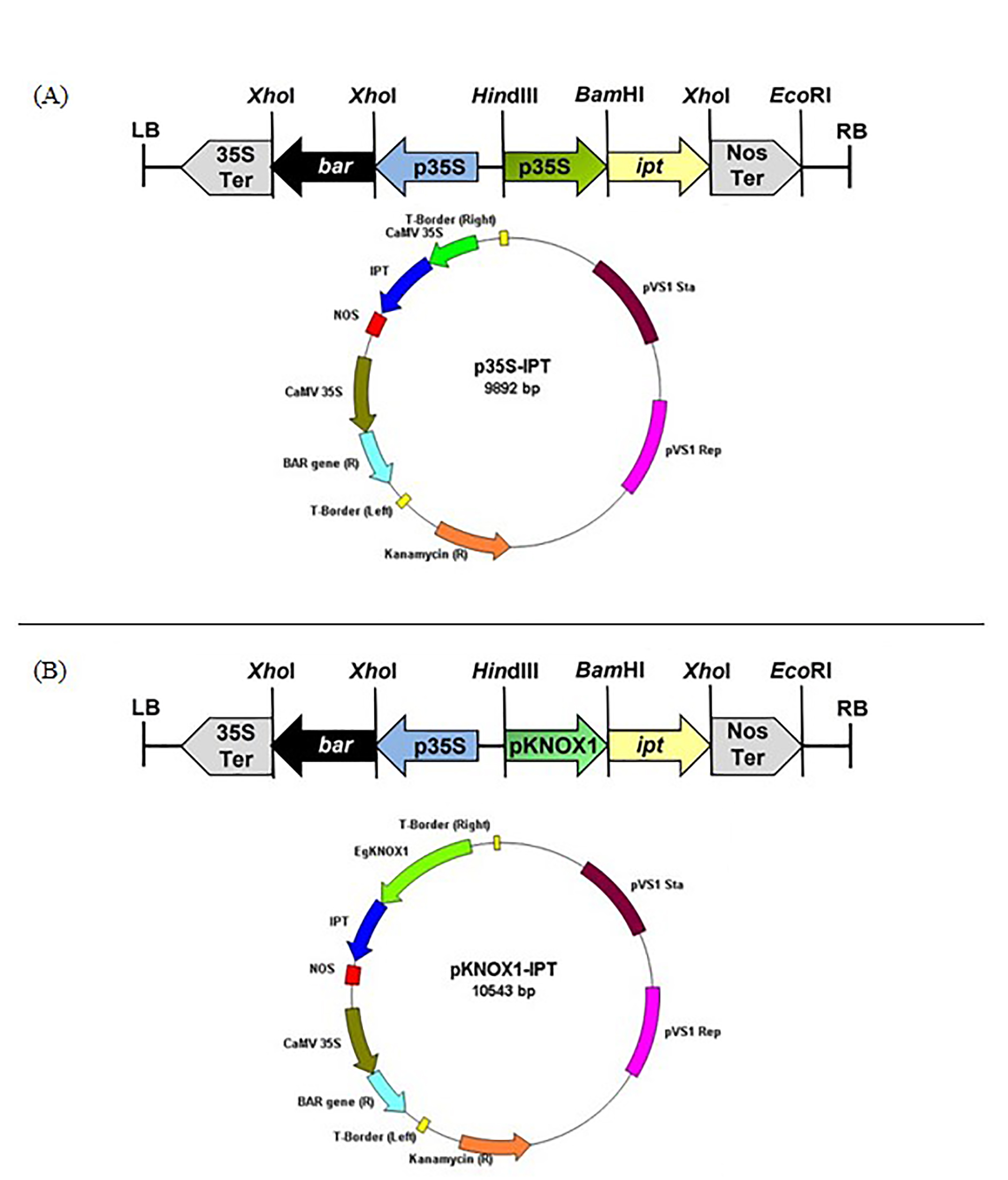

Supplement: Supplementary Figure 1 — Schematic diagrams of expression vectors under the control of CaMV 35S (A) and KNOX1 (B) promoter. [file Image_1.jpeg]

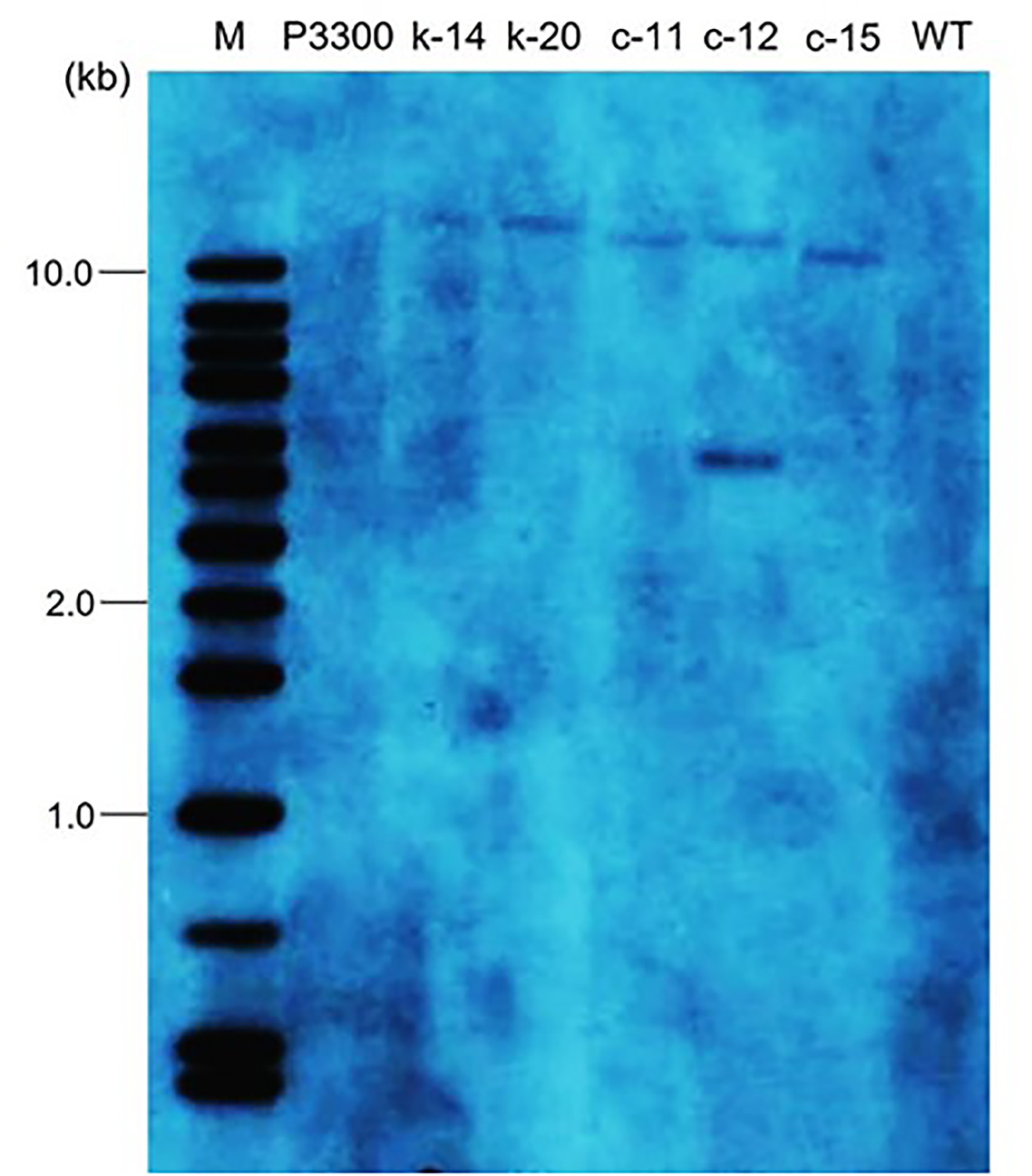

Supplement: Supplementary Figure 2 — Identification of representative of transgenic P. pellucida plant lines carrying integrated T-DNA resulted by PCR and Southern blotting analysis digested with BamHI and XhoI. Molecular markers are indicated on the left. Lane M, molecular size marker 1 kb; Lane 1, transgenic control plant without ipt gene (P3300); Lanes 2–3, transgenic ipt plants with KNOX1 promoter (k-14, k-20); Lanes 4–6, transgenic ipt plants with 35S promoter (c-11, c-12, c-15); Lane 7, wildtype plant. [file Image_2.jpeg]

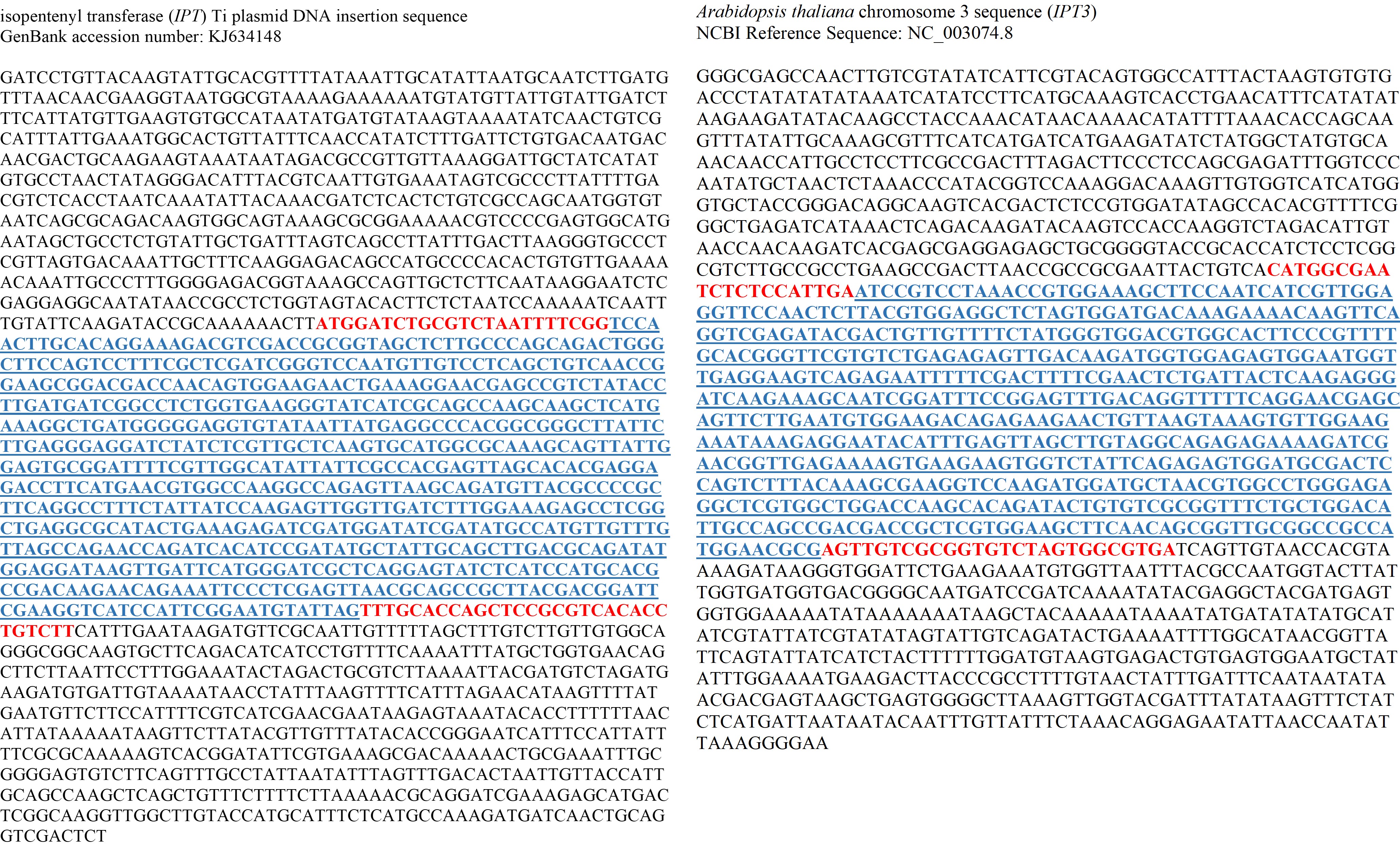

Supplement: Supplementary Figure 3 — Nucleotide sequence and conserved region of IPT and IPT3 gene. [file Image_3.jpg]
