# Supplementary material for: Stable and reproducible expression of bacterial ipt gene under the control of SAM-specific promoter (pKNOX1) with interference of developmental patterns in transgenic Peperomia pellucida plants
Source: Front Plant Sci. 2022 Sep 27;13:984716. doi: 10.3389/fpls.2022.984716 (PMC9551203; doi:10.3389/fpls.2022.984716)
Supplement: Supplementary Table 1 — List of primer sequences used for PCR and qPCR analysis. [file Table_1.docx]

**Table S1.** List of primer sequences used for PCR and qPCR analysis.

| **Gene** | **Primer** | **Sequence (5’ to 3’)** | **Amplicon size (bp)** | **Annealing temp (֯C)** |
| --- | --- | --- | --- | --- |
| **bacterial *ipt*** | Forward | ATGAGCCCAGAACGACGCCCG | 129 | 55 |
|  | Reverse | TCAAATCTCGGTGACGGGCAGG |  |  |
| **plant *ipt3*** | Forward | CATGGCGAATCTCTCCATTGA | 123 | 55 |
|  | Reverse | AGTTGGAACCTCCAACGATGA |  |  |
| ***bar*** | Forward | ATGAGCCCAGAACGACGCCCG | 94 | 50 |
|  | Reverse | TCAAATCTCG GTGACGGGCAGG |  |  |
| ***ACT2*** | Forward | TTGTTTGTTTCATTTCCCTTTTTG | 112 | 55 |
|  | Reverse | GCAGACGTAAGTAAAAACCCAGAGA |  |  |
